# Factors associated with refractory autoimmune necrotizing myopathy with anti-signal recognition particle antibodies

**DOI:** 10.1186/s13023-020-01431-7

**Published:** 2020-07-08

**Authors:** Yawen Zhao, Wei Zhang, Yilin Liu, Zhaoxia Wang, Yun Yuan

**Affiliations:** Department of Neurology, Peking University First Hospital, No. 8 Xishku Street, Xicheng District, Beijing, 100034 PR China

**Keywords:** Autoimmune necrotizing myopathy, Anti-signal recognition particle antibodies, Refractory myositis, Thigh MRI, B cell activating factor receptor

## Abstract

**Background:**

Autoimmune necrotizing myopathy with anti-signal recognition particle antibodies (ANM-SRP) is regarded as refractory myositis, whereby some patients respond poorly to conventional immunosuppression and require B cell depletion treatment. This study aimed to evaluate factors associated with refractory ANM-SRP.

**Results:**

Clinical and pathological data from 48 patients with ANM-SRP were collected. We followed up clinical symptoms and image changes over 12 months. Univariate and multivariate analyses were undertaken to determine the associations between variables of interest and poor response to therapy. Refractory ANM-SRP appeared in 32.5% of patients who showed no or minimal improvement after 12 months of steroid therapy. The clinical risk factors for refractory patients were being male (OR, 19.57; *P* < 0.001), severe muscle weakness (OR, 7.51; *P* < 0.001) and concurrent interstitial lung disease (OR, 39.70; *P* < 0.001). The imaging refractory-related factor was the fatty infiltration rate of thigh muscles over 3 months (*P* = 0.022) and the pathological factor associated with refractory ANM-SRP was the high expression of B cell activating factor receptor (BAFF-R) in muscle (*P* = 0.036).

**Conclusion:**

Being male, severe muscle weakness, concurrent interstitial lung disease, quick development of muscle fatty infiltration and more BAFF-R and B lymphocyte infiltration in muscle indicate a poor response to immunosuppressive therapy in patients with ANM-SRP.

## Background

Autoimmune necrotizing myopathy with anti-signal recognition particle antibodies (ANM-SRP) is a subtype of a group of idiopathic inflammatory myopathies characterized by the presence of anti-SRP antibody in serum [1]. It is often treated as refractory myositis because of its poor response to immunosuppressive agents including glucocorticoids and second-line immunosuppressive therapy [2, 3], especially in patients with other myositis-specific autoantibodies [4, 5] or other autoimmune diseases [6], often requiring a combination of medications [2, 7]. Japanese studies also used glucocorticoids as basic drugs, 77% of which required additional immunosuppressants [8]. B lymphocyte depletion therapy (rituximab) has been shown to be beneficial in refractory patients, which suggests that the proliferation and differentiation of B lymphocytes play an important role in the pathogenesis of ANM-SRP [3, 9, 10].

Several investigations demonstrated that early-onset disease, severe myasthenia, dysphagia, and muscular atrophy, combined with concurrent interstitial lung disease (ILD) may be risk factors for poor prognosis of ANM-SRP [2, 8]. Additionally, patients with a chronic disease course often show more severe myasthenia symptoms and muscular atrophy, as well as poorer treatment response and prognosis [11]. In our previous muscle MRI study of 12 patients, we found that four patients presented with severe thigh muscle fatty infiltration of posterior group and muscle fatty infiltration was one factor for refractory disease [12]. However, the proportion of patients with refractory ANM-SRP and pathological indicators associated with response to conventional immunosuppression are lacking due to few systematic follow-up studies. In this study, we therefore sought to evaluate the clinical and pathological factors influencing the therapeutic effect of conventional immunosuppression in a large cohort of Chinese patients with ANM-SRP.

## Methods

### Patient registry

This was a single-center, retrospective, observational cohort study including 48 Chinese patients who were diagnosed with ANM-SRP according to clinical, serological, and pathological criteria. Demographic data, clinical features, and initial drug treatment information were collected (Supplementary Materials). Written informed consent was obtained from all patients (or an appropriate family member where the patient was unable to provide consent). The procedures were performed in accordance with the ethical standards of the responsible committee on human experimentation and approved by the Institutional Review Board.

### Serum myositis antibody test

An immunoblot kit was used to determine the serum antibodies to SRP by detecting the 54kD subunit. The membrane strips were pretreated, incubated with patient serum, and subjected to enzyme binding. The results were scanned using EUROlineScan software, and were recorded as negative, weak positive (+), positive (++), and strong positive (+++). All the patients recruited were strong positive (+++).

### Muscle MRI

Thirty-six patients underwent bilateral thigh MRI (tMRI) (3.0 T, GE 1.5 Sigma Twin Speed; GE Healthcare, Waukesha, WI, USA). Axial T1-weighted MRIs were used to evaluate the degree of fatty infiltration according to the modified Mercuri scale (0–5 scale; from normal appearance to complete fatty infiltration). Axial short T1 inversion recovery sequences were used to assess the degree of edema (0–5 scale, from normal to moderate intrafascicular global edema) [11]. Edema and fatty infiltration scores were calculated in the gluteus maximus at the pelvic level and thigh muscles (vastus intermedius, vastus medialis, vastus lateralis, rectus femoris, biceps femoris, semitendinosus, semimembranosus, adductor magnus, sartorius, long adductor, and gracilis) at the mid-thighs. Additionally, we compared the thigh MRI of 36 patients with 36 controls (autoimmune necrotizing myopathy patients with negative anti-SRP antibody) to observe the damage in the tMRI. Total edema and the degree of fatty infiltration were compared before and after treatment as an indicator of therapeutic effect.

### Muscle pathology

Muscle biopsies were taken from the biceps brachii or quadriceps femoris of all patients. Serial frozen sections were stained with hematoxylin and eosin (HE), modified Gomori trichrome, periodic acid-Schiff, oil red O, adenosinetriphosphate (ATP) enzyme (pH 4.5 and 10.8), NADH-tetrazolium reductase, succinate dehydrogenase (SDH), and cytochrome C oxidase (COX) stains. The sections were immunohistochemically stained with primary antibodies against human CD3, CD4, CD8, CD20, CD68, major histocompatibility complex class-I (MHC-I), membrane attack complex (MAC), dystrophin, sarcoglycans, and dysferlin.

### BAFF and BAFF-R measurement

To further clarify the role of B lymphocytes in the therapeutic effect on ANM-SRP, immunohistochemical staining of B cell activating factor (BAFF), B cell activating factor receptor (BAFF-R) and CD19 was performed in 29 patients. The positive cellular expression of BAFF, BAFF-R and CD19-positive cells (numbers of positive cells / numbers of muscle fibers) was calculated per high-power microscopic field: 10 high-power microscopic fields (400×) were randomly selected from each section and the numbers of staining-positive cells and muscle fibers were counted under each high-power microscopic field (those with more than ½ area were included and those with less than ½ area were omitted). The average value of 10 high-power fields was calculated. Correlation of the positive cellular expression of BAFF, BAFF-R, CD19-positive cells was analyzed.

Additionally, muscle tissues of 14 patients with ANM-SRP and four healthy controls were sampled for semi-quantitative analysis of BAFF and BAFF-R. Radioimmunoprecipitation assay (RIPA) lysate (including protease inhibitor and phosphatase inhibitor) was added to the tissue samples for homogenization and the protein was extracted after lysis on ice for 30 min. An equal amount of protein (80 μg) was suspended in loading buffer, denatured at 100 °C for 5 min, and loaded on an SDS-PAGE gel. After being electrophoresed in 10% polyacrylamide gel, transferred electrophoretically to nitrocellulose membrane, the membrane was blocked with non-fat milk buffer for 1 h and then incubated with the primary antibodies overnight at 4 °C. The primary antibodies were IgG-specific for BAFF (Abcam, ab16081, 1:500), BAFF-R (Abcam, ab5965, 1:500), and actin (Abcam, ab8226, 1:3000). After three 5-min washes (20-mmol/L Tris, pH 7.6, 8 g sodium chloride, 0.05% Tween-20), blots were incubated for 50 min with horseradish peroxidase-conjugated goat anti-rabbit IgG or rabbit anti-rat IgG (1:5000). After washing, bound IgG was detected auto-radiographically by enhanced chemiluminescence (Quantity One v.4.6.2).

### Clinical follow-up and efficacy evaluation

The modified Rankin Scale score (mRS) was evaluated at 3, 6, and 12 months after treatment to evaluate the therapeutic effect on the basis of glucocorticoid therapy combined with other immunosuppressive agents or intravenous immunoglobulins (IVIg) in the study period. Patients were divided into non-refractory and refractory groups. If patients’ muscle strengths returned to normal or was close to normal, the mRS scores were 0–2 points and those patients were placed in the non-refractory group. If there was still obvious limb weakness after 12 months of immunotherapy, the mRS scores were 3–5 points, or patients had a relapse, then the patients were placed into the refractory group [8, 13]. The relapse criteria were that the serum creatine kinase(CK) returned to above the patient’s baseline level, rose to more than three times the reference upper limit, and limb weakness worsened again (excluding other causes such as exercise, electrolyte disturbance, thyroid dysfunction, or drug induction) [9].

Twenty-five patients with ANM-SRP were followed up by tMRI. We monitored the muscle fatty infiltration and edema changes by tMRI for 3, 6, 12, 18, and 24 months after treatment. Imaging indicators were the average change rates of thigh muscle fatty infiltration and edema (the scale gap of tMRI / interval time between before and after treatment) for 3, 6, 12, 18, and 24 months after treatment.

### Statistical analysis

All analyses were performed using SPSS 17.0 software. Single factor and binary logistic regression analyses were used to compare the clinical, tMRI changes and pathological features between the two groups. The Mann–Whitney *U* test or Kruskal–Wallis test was used for the continuous variables and Chi-square test for categorical variables. Multivariate logistic regression analysis was performed on related predictors for refractory disease. Explanatory variables were selected using a liberal criterion (*p* < 0.10) for inclusion in the multivariate regression model. For all statistical analyses, significance was accepted as *p* < 0.05.

## Results

The follow-up study was completed in 40 out of 48 enrolled patients, with a loss-to-follow-up rate of 16.67%. The mean follow-up duration was 3.85 ± 2.49 years (12 months to 9 years). After 12 months of treatment, 27 patients (67.5%) had normal or nearly normal muscle strength and an mRS 0–2 (the non-refractory group) and 13 patients (32.5%) still had muscle weakness and an mRS 3–5 (the refractory group).

### Clinical refractory related factors: male, severe muscle weakness, concurrent ILD

In our cohort, the ratio of male to female participants was 14:34 and the cohort comprised 6 teenagers and 42 adults. The mean onset age was 40.9 ± 17.2 years old and the time to admission was 6 (4, 18) months. The numbers of patients with acute, subacute, and chronic disease were 5, 24, and 19, respectively. According to the Medical Research Council (MRC) classification, 18 patients (37.5%) had severe weakness (MRC 1–2/5), 20 patients (41.7%) moderate weakness (MRC 3/5), and 10 patients (20.8%) mild weakness (MRC 4–5/5). The severe weakness appeared in the lower limbs of 24 patients (50.0%), in the upper limbs of 6 patients (12.5%), and in all limbs of 18 patients (37.5%). Additional clinical baseline data can be seen in the Supplementary Material.

The mean onset age in the non-refractory group and refractory group were 37.26 ± 18.06 and 50.77 ± 15.77 years old, respectively (*P* = 0.029). The proportions of weight lost after the onset of disease were 40.7% in the non-refractory group and 76.9% in the refractory group (*P* = 0.032). Furthermore, the percentage of ILD was 22.2% in the non-refractory group and 69.2% in the refractory group (*P* = 0.004) and the proportion of patients initially treated with methotrexate was 59.3% for the non-refractory group and 23.1% for the refractory group (*P* = 0.032; Table 1). Binary logistic regression analysis verified that the risk factors for refractory patients were being male (OR = 19.57, 95%CI = 1.49–256.53), severe muscle weakness (OR = 7.51, 95%CI = 41.03–54.88), and the presence of ILD (OR = 39.70, 95%CI = 3.04–518.38; Table 2).

**Table 1:** Clinical indicators between refractory and non-refractory group

	**Non-refractory group (27)**		**Refractory group (13)**		***P***
	Total(n)	Proportion(%)	Total(n)	Proportion(%)	
**Clinical syndromes**					
Gender(male: female)	5:22	18.5	6:7	46.2	0.067
Cervical flexion weakness	22	81.5	11	84.6	0.807
Severe myasthenia	9	33.3	8	61.5	0.091
Dysphagia	9	33.3	5	38.5	0.750
Dysmasesia	7	25.9	1	7.7	0.177
Myalgia	10	37.0	4	30.8	0.697
Amyotrophy	11	40.7	6	46.2	0.746
Weight lost	11	40.7	10	76.9	0.032
**Auxiliary examination**					
ILD	6	22.2	9	69.2	0.004
Elevated ESR	5	18.5	5	38.5	0.872
Elevated ANA	12	44.4	8	61.5	0.311
Combined with anti-Ro-52 antibody	7	25.9	5	38.5	0.418
**Initial therapy**					
GC	3	11.1	4	30.8	0.132
GC + IVIg+MTX	10	37.0	3	23.1	0.390
GC + MTX	6	22.2	1	7.7	0.269
GC + IVIg	5	18.5	3	23.1	0.744
GC + OI	4	14.8	5	38.5	0.098
OI + MTX	16	59.3	3	23.1	0.032
OI + IVIg	15	55.6	6	46.2	0.588

*ILD* Interstitial lung disease, *GC* Glucocorticoid, *MTX* Methotrexate, *IVIg* Intravenous immunoglobulin, *OI* Other immunosuppressant

**Table 2:** Binary logistic analysis of clinical indicators between refractory and non-refractory group

**Indicator**	**OR**	**95%CI**	***P***
Gender(male)	19.57	1.49–256.53	0.024*
Severe muscle weakness	7.51	41.03–54.88	0.047*
With ILD	39.70	3.04–518.38	0.005*

*ILD* Interstitial lung disease

*P<0.05

### Imaging refractory-related factors: quick development of muscle fatty infiltration

Thirty-six patients underwent tMRI, which showed fatty infiltration in 29 patients (80.6%) and edema in 32 patients (88.9%). The fatty infiltration and edema mainly affected the gluteus maximus, adductor magnus, semimembranosus, semitendinosus and the long head of the biceps both in the refractory and non-refractory groups. There was no statistically significant difference between the thigh MRI of patients with ANM-SRP and 36 ANM patients with negative anti-SRP antibody.

**Fig. 1:**
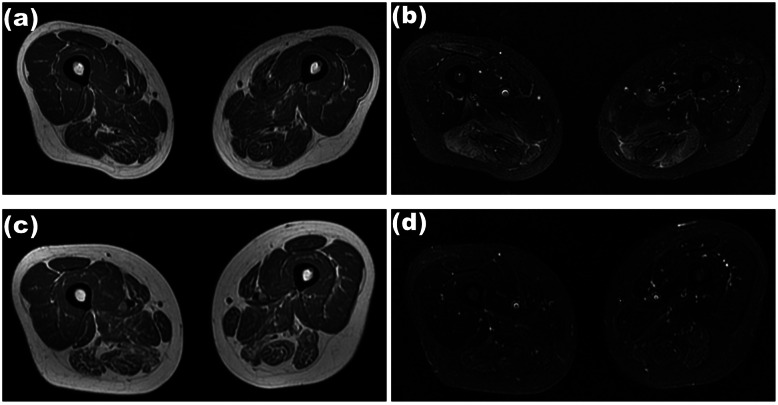
Thigh MRI of refractory ANM-SRP. Thigh MRI showed mild fatty infiltration (**a**; T1-weighted MRI) and focal edema (**b**; Short T1 inversion recovery sequences) in thigh muscle before treatment, the fatty infiltration aggravated (**c**) and muscle edema alleviated (**d**) after immunotherapy

Thigh MRI follow-up showed an increasing rate of fatty infiltration at 3, 6, 12, 18, and 24 months with 3.44, 1.45, 0.68, 0.41, and 0.11, respectively (Fig. 1). The reducing rate of edema at 3, 6, 12, 18, and 24 months was 2.37, 2.72, 1.07, 0.55, and 0.57, respectively. The mean fatty infiltration rate of the thigh muscles at 3 months was higher in the refractory group than in the non-refractory group (*P* = 0.022). However, there was no statistically significant difference between the two groups in the increasing rate of fatty infiltration and the reducing rate of edema at the remaining periods (Table 3).

**Table 3:** Imaging indicators between refractory and non-refractory group

**tMRI**	**Average change rates**	**Non-refractory group (16)**	**Refractory group (9)**	***P***
Fatty infiltration	3 months	2.64	5.55	0.022*
	6 months	1.37	1.60	0.118
	12 months	0.77	0.59	0.305
	18 months	0.43	0.38	0.534
	24 months	0.14	0.17	0.176
Edema	3 months	3.69	2.17	0.240
	6 months	2.80	2.56	0.206
	12 months	1.18	1.17	0.627
	18 months	0.99	0.70	0.431
	24 months	0.64	0.60	0.386

*tMRI* Thigh MRI

*P<0.05

### Pathological refractory-related factors: high expression of BAFF and BAFF-R in muscle

**Fig. 2:**
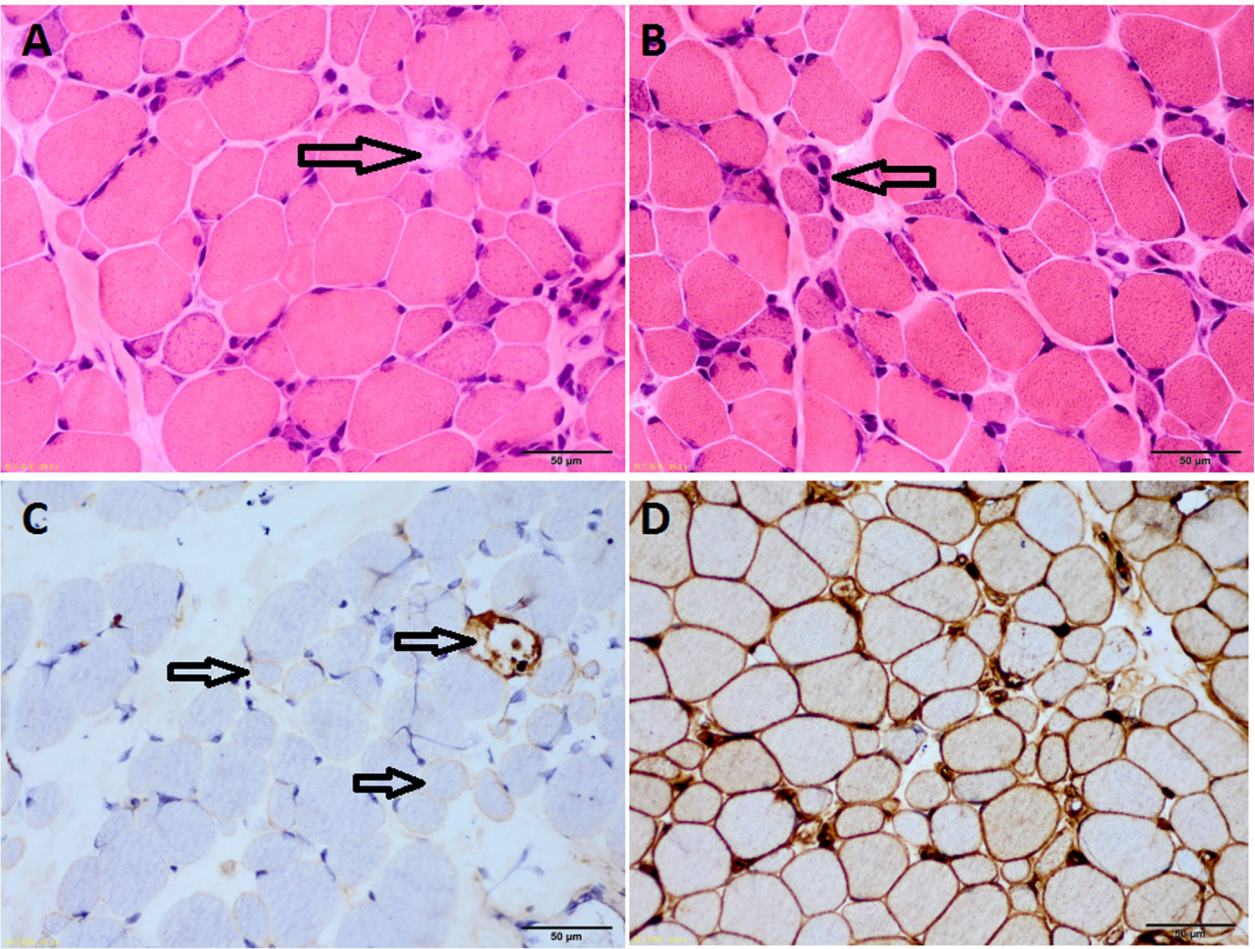
Muscle pathology of ANM-SRP (× 400). Muscle fibers show necrosis (**a**, arrow) and regeneration (**b**, arrow), with little inflammatory cell infiltration (H&E staining); MAC staining (**c**) showed MAC deposition of necrotic muscle fiber and non-necrotic sarcolemma (arrow) and MHC-I staining (**d**) showed positive expression of sarcolemma and partial cytoplasm, accompanied by decreased capillaries

Muscle biopsies revealed a large variation in fiber size in all patients except two patients with terminal changes. Muscle fiber hypertrophy appeared in 10 patients (22.7%). Muscle fiber necrosis with myophagocytosis appeared in 40 patients (90.9%) and muscle fiber regeneration appeared in 43 patients (97.7%). CD68^+^ macrophages appeared in necrotic fibers in the perimysium in 41 patients (93.2%). Ragged blue fibers were found in three patients using SDH staining (7.5%), and COX-negative muscle fibers were found in six patients using Cox staining (15%). Mild-to-moderate connective tissue proliferation appeared in 13 patients (29.5%). Perivascular lymphocyte infiltration in the perimysium was observed in nine patients (20.5%), while CD3^+^, CD4^+^ and CD8^+^ lymphocytes appeared in 22 (50%), 26 (59.1%) and 23 (52.3%) patients, respectively. CD20^+^ lymphocytes appeared in four patients (9.1%) and CD19^+^ lymphocytes appeared in 21 patients (72.4%) of 29 tested patients. MAC deposition in muscle fibers was seen in 32 of 37 patients (86.5%). MHC-I positive myofibrils appeared in 37 of 40 patients (92.5%; Fig. 2).

Pathological indicators like muscle fiber necrosis, regeneration, atrophy, hypertrophy, connective tissue proliferation, infiltration of CD3^+^, CD4^+^, CD8^+^, and CD20^+^ lymphocytes, infiltration of CD68^+^ macrophages, MAC deposition, and MHC-I positive expression were not significantly different between the refractory and non-refractory groups.

**Fig. 3:**
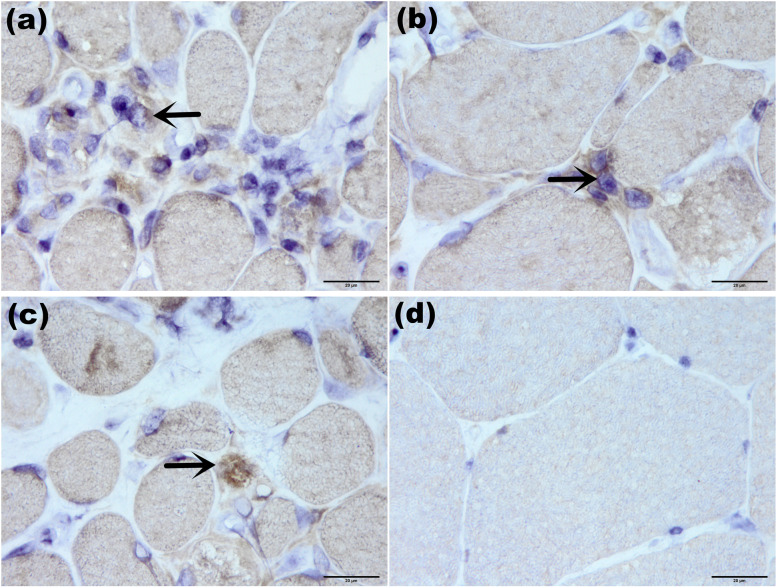
BAFF staining of ANM-SRP(×1000). BAFF was positively expressed on the surface of inflammatory cells in endomysium (**a**, **b**, arrow) and on the surface of inflammatory cells invading necrotic muscle fibers (**c**, arrow). No BAFF positive expression was found in healthy control (**d**)

**Fig. 4:**
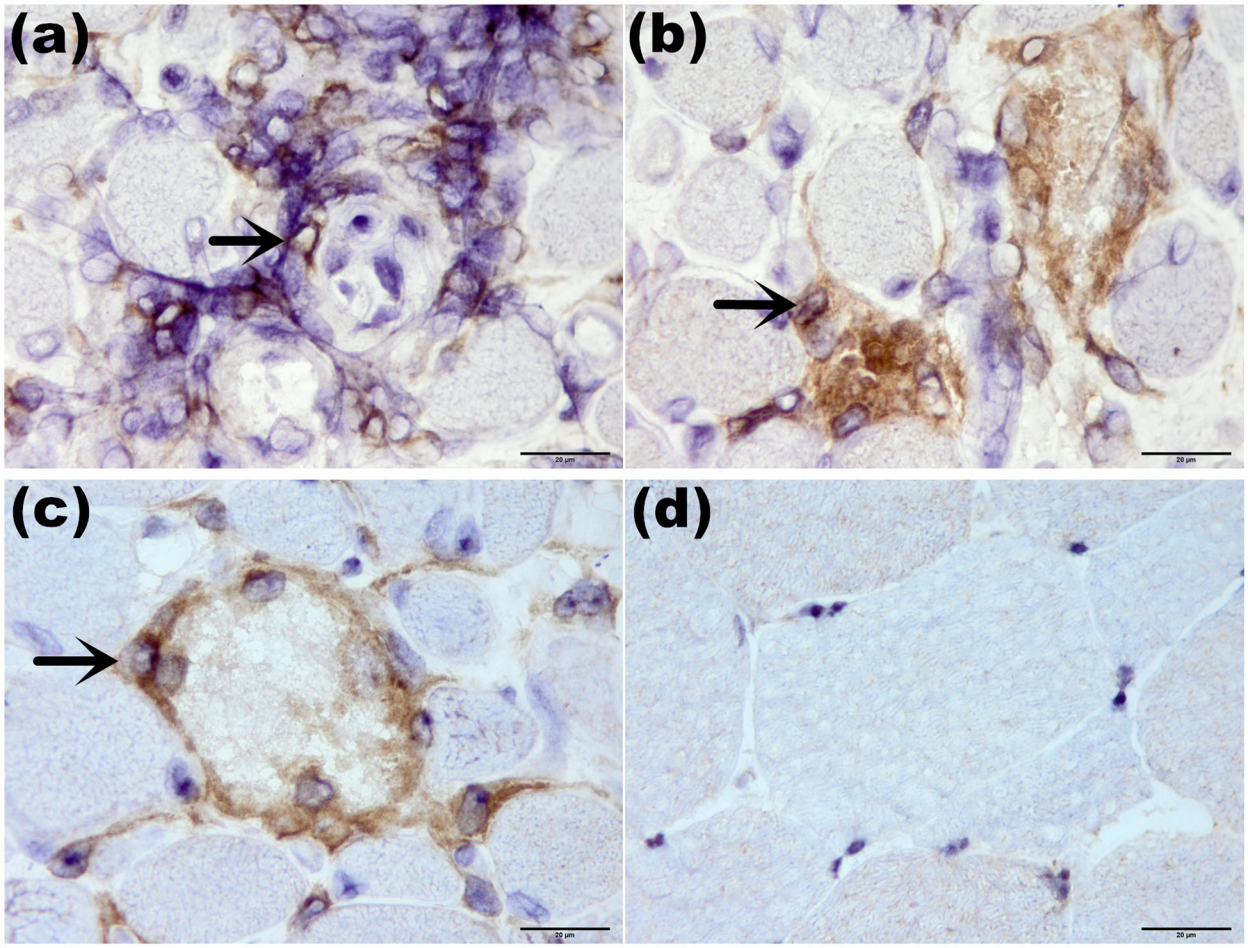
BAFF-R staining of ANM-SRP(× 1000). BAFF-R was positively expressed on the surface of inflammatory cells surrounding muscle fibers (**a**, arrow) or on the surface of inflammatory cells surrounding and invading necrotic muscle fibers (**b**, **c**, arrow). No BAFF-R positive expression was found in healthy controls (**d**)

**Fig. 5:**
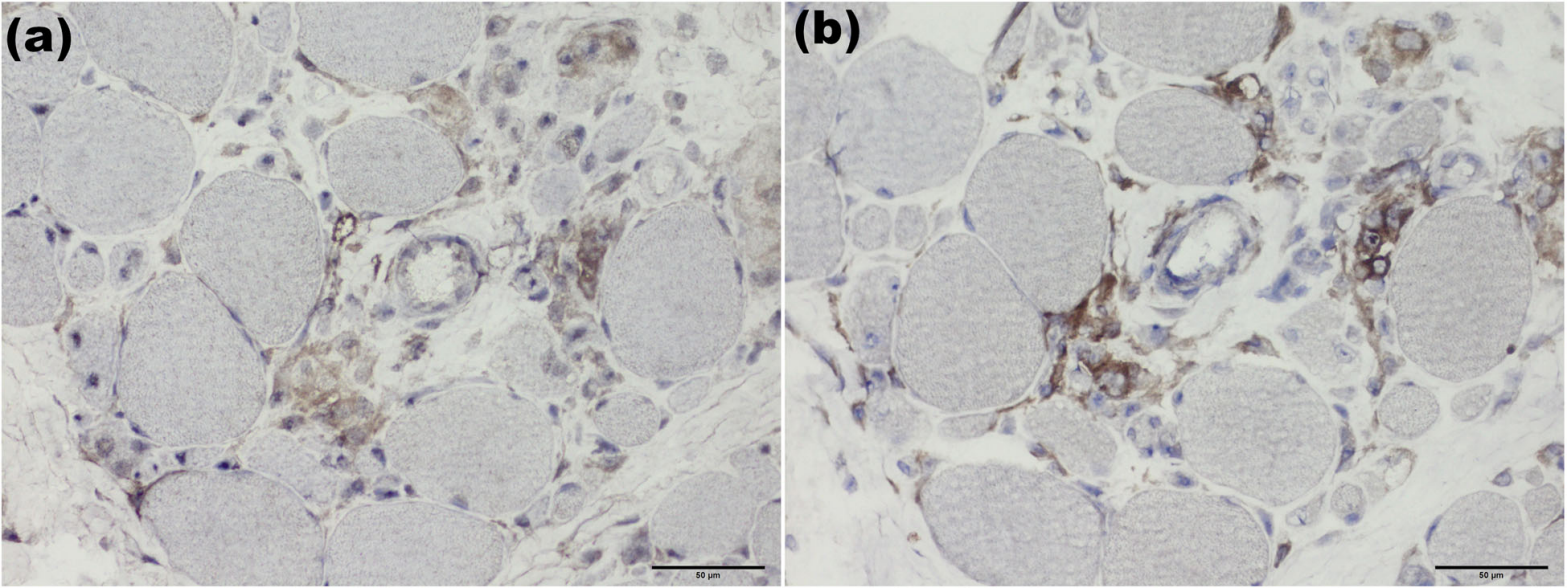
The expression sites of BAFF-R (**a**, × 400) were highly overlapping compared with CD19 (**b**, × 400)

BAFF staining revealed that 10 of 29 patients (34.5%) with positive deposition in necrotic tissue regenerated muscle fibers and individual lymphocytes (Fig. 3). Positive BAFF-R expression was found in 24 of 29 patients (82.8%), mainly expressed in necrotic muscle fibers, muscle perimysium, muscle underwear and by lymphocytes infiltrating around blood vessels (Fig. 4). The expression level of CD19^+^ lymphocytes overlapped with BAFF-R (Fig. 5). Spearman correlation tests showed a correlation between BAFF-R and CD19 (*R* = 0.818, *P* < 0.001).

**Fig. 6:**
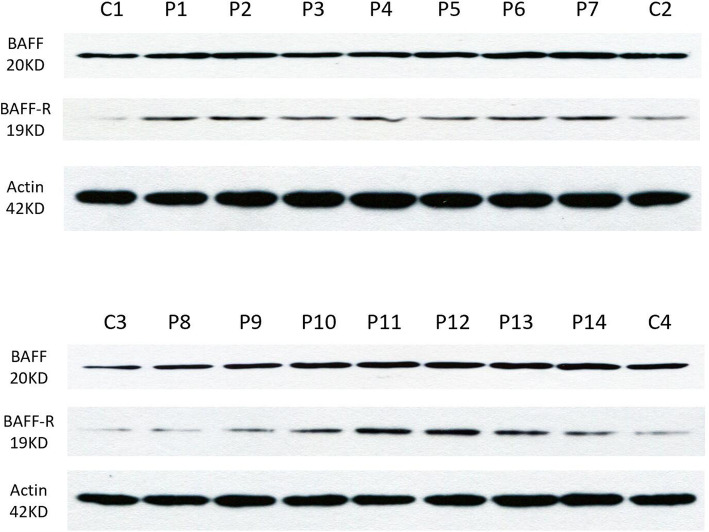
Western bolt of BAFF and BAFF-R in skeletal muscle of ANM-SRP. The results showed that the expression of BAFF-R in skeletal muscle of the patients was significantly higher than that of the healthy control. BAFF were expressed in skeletal muscle of both patients and healthy control

The positive cellular expression of BAFF-R in muscles was 0.27 ± 0.14 for the non-refractory group and 0.42 ± 0.23 for the refractory group (*P* = 0.036). The positive cellular expression of CD19 in skeletal muscle was 0.18 ± 0.08 for the non-refractory group and 0.36 ± 0.21 for the refractory group (*P* = 0.002). There was no statistically significant difference in the expression of BAFF (Table 4). The western blots of BAFF and BAFF-R in skeletal muscle of patients and healthy controls also showed that BAFF-R expression in patients’ skeletal muscle was significantly higher than that of the healthy controls. BAFF was expressed in skeletal muscle of both patients and healthy controls (Fig. 6).

**Table 4:** Pathology indicators between refractory and non-refractory group

**Positive cellular expression**	**Non-refractory group(19 cases)**	**Refractory group(10 cases)**	***P***
BAFF	0.06 ± 0.03	0.06 ± 0.05	0.542
BAFF-R	0.27 ± 0.14	0.42 ± 0.23	0.036*
CD19	0.18 ± 0.08	0.36 ± 0.21	0.002*

*P<0.05

## Discussion

This study examined the factors contributing to poor response to immunosuppressive agents in ANM-SRP patients. We found that male patients with ANM-SRP have a poorer prognosis, but this conclusion may still need to be confirmed in a larger sample. Our previous study followed up three child patients with good prognosis, all of whom were female [14], while most studies had not found a correlation between gender and refractory disease [8, 13]. We also found that severe muscle weakness before the initiation therapy indicated a poor response which has also been reported by Aggarwal and colleagues in a cohort of 25 patients with ANM-SRP [15]. More than 90% of patients in a Brazilian study showed mild and moderate muscle weakness, and the refractory rate was 42.9% [16]. Whether there is a racial difference between the degree of limb weakness and the therapeutic effect needs further analysis. In the refractory group, the proportion of patients with ILDs was high (34.8%) and its incidence was close to that of Brazilian patients (36%) [16], which is higher than the 25% reported in Japan, Europe and America [8, 17, 18]. The combination of ILDs is an important factor affecting patients’ quality of life and prognosis, while the use of mycophenolate or tacrolimus may improve the therapeutic effect in patients with the combination of ILDs [18, 19].

Our study added skeletal muscle MRI to monitor disease activity and severity and was used for long-term follow-up of patients. The fatty infiltration exacerbation of skeletal muscle MRI in the early treatment period often suggests a refractory disease. Similar conclusions can be seen in other MRI studies of skeletal muscle in idiopathic inflammatory myopathy [20]. A previous study showed that muscle fatty infiltration may be closely related to the prognosis of ANM-SRP and is often a marker of glucocorticoid resistance or refractory treatment [12, 21]. Therefore, it is recommended that patients with ANM-SRP should have skeletal muscle MRIs within 3 to 6 months after treatment initiation to monitor the efficacy of immunotherapy and the progression of the disease. For patients with early fatty infiltration changes, immunotherapy should be strengthened and more frequent follow-up should be conducted. Whether the approach preventing fatty infiltration of skeletal muscle will reduce the poor response to treatment warrants further studies.

In this study, we observed that BAFF-R expression was upregulated in the muscle of patients with ANM-SRP. BAFF, a member of the tumor necrosis factor (TNF) ligand family, has been shown to play an important role in the homeostasis and autoimmunity of B lymphocytes [22]. BAFF-R is expressed in B lymphocytes and binds with high specificity to BAFF. BAFF-BAFFR interaction plays a leading role in maintaining peripheral mature and immature B lymphocyte pool and is the main receptor required for BAFF-mediated survival of mature B lymphocytes [23]. Previous studies have detected the upregulation of BAFF and BAFF-R in the serum or muscle of dermatomyositis, polymyositis, and anti-synthase antibody syndrome patients [24, 25], and its level correlated with the activity of idiopathic inflammatory myopathy [24]. Patients with ANM-SRP had high BAFF-R and CD19 expression, and the refractory patients had significantly higher BAFF-R and CD19 expression than non-refractory patients, which suggests that BAFF and its receptors may cause muscle fiber injury and lymphocyte proliferation. CD19 and BAFF-R expression in skeletal muscle might be effective pathological indicators to predict the treatment effects in patients. A number of small sample studies suggest that removal of B cells is effective for treating refractory ANM-SRP [26], while this study also provided pathological evidence for the use of B lymphocyte depletion drugs and suggested that BAFF inhibitors [27] may be effective in the treatment of patients with ANM-SRP.

Our study has several limitations that require comment. First, it was a single-center and hospital-based study that focused on urban patients only and therefore could not reveal the true situation of patients with ANM-SRP in rural areas or remote regions. Patients who did not seek treatment or who died before arriving at the hospital were not included in the study. Furthermore, it was a retrospective, observational cohort study, with initial differences in therapy in some patients, which may have affected the results, so we used a multivariate logistic regression analysis to reduce this interference. Prospective studies with a larger sample are needed in the future to verify our conclusions.

## Conclusion

The present study examined a multitude of clinical and pathological factors that may be associated with the refractory ANM-SRP. We found that BAFF-R expression in skeletal muscle of ANM-SRP was upregulated. Being male, severe muscle weakness, concurrent ILD, quick development of muscle fatty infiltration, and higher BAFF-R and B lymphocyte muscle infiltration may be the factors associated with refractory ANM-SRP.

## Supplementary information

**Additional file 1: Supplementary materials.** Demographic and clinical features of patients with ANM-SRP.

## Data Availability

The patients data used in this study are included in this publish article and its supplementary files.

## Abbreviations

ANM-SRP: Autoimmune necrotizing myopathy with anti-signal recognition particle antibodies
BAFF: B cell activating factor
BAFF-R: B cell activating factor receptor
CK: Creatine kinase
ILD: Interstitial lung disease
tMRI: thigh MRI
HE: Hematoxylin and eosin
ATP: Adenosinetriphosphate
SDH: Succinate dehydrogenase
COX: Cytochrome C oxidase
MHC-I: Major histocompatibility complex class -I
MAC: Membrane attack complex
IVIg: Intravenous immunoglobulins
mRS: The modified Rankin Scale score
MRC: Medical Research Council
