# Supplementary material for: Factors associated with refractory autoimmune necrotizing myopathy with anti-signal recognition particle antibodies
Source: Orphanet J Rare Dis. 2020 Jul 8;15:181. doi: 10.1186/s13023-020-01431-7 (PMC7341563; doi:10.1186/s13023-020-01431-7)
Supplement: Supplementary file 1 — Additional file 1: Supplementary materials. Demographic and clinical features of patients with ANM-SRP. [file 13023_2020_1431_MOESM1_ESM.docx]

**[Supplementary](D:/%E8%A3%85%E6%9C%BA%E8%BD%AF%E4%BB%B6/Youdao/Dict/7.5.0.0/resultui/dict/?keyword=supplementary)[materials](D:/%E8%A3%85%E6%9C%BA%E8%BD%AF%E4%BB%B6/Youdao/Dict/7.5.0.0/resultui/dict/?keyword=materials): Demographic and clinical features of patients with anti-SRP antibody myopathy**

| **Gender** | Male | 14 |
| --- | --- | --- |
|  | Female | 34 |
| **Onset age(years old)** | | 40.9±17.2 |
| **Time from diagnosis to onset(month)** | | 6（4,18） |
| **Onset form** | acute N(n%) | 5（10.4%） |
|  | subacute N(n%) | 24（50%） |
|  | chronic | 19（39.6%） |
| **Cervical weakness N(n%)** | | 37（77.1%） |
| **Limb weakness** | proximal N(n%) | 47（97.9%） |
|  | distal N(n%) | 1（2.1%） |
| **The median strength** | Proximal of UL | 4（2，5） |
|  | Distal of UL | 5（4，5） |
|  | Proximal of LL | 3（2，5） |
|  | Distal of LL | 5（4，5） |
|  | Neck flexor muscles | 3（1，5） |
| **Dysphagia N(n%)** | | 37（77.1%） |
| **Myalgia N(n%)** | | 17（35.4%） |
| **Muscle atrophy N(n%)** | | 19（39.6%） |
| **Dysmasesia N(n%)** | | 10（20.8%） |
| **Exertional dyspnea N(n%)** | | 5（10.4%） |
| **Dysarthria N(n%)** | | 2（4.2%） |
| **Weight loss N(n%)** | | 24（50%） |
| **Statin explosure N(n%)** | | 8（16.7%） |
| **The residual stomach cancer N(n%)** | | 1（2.1%） |
| **Sjogren's syndrome** | | 2（4.2%） |
| **Serum CK(IU/L)** | | 4672.0 ± 2915.4 |
| **ESR↑n/N(n%)** | | 14/48（29.2%） |
| **ANA>1:320 n/N(n%)** | | 22/46(47.8%) |
| **EMG** | Myogenic damage n/N(n%) | 44/48（91.7%） |
|  | Spontaneous potential n/N(n%) | 28/48（58.3%） |
| **Abnormal NCV n/N(n%)** | | 9/48（18.8%） |
| **ILD n/N(n%)** | | 16/46（34.8%） |
| **Pulmonary dysfunction n/N(n%)** | | 6/18（33.3%） |
| **Myositis antibody spectrum** | Isolated SRP (+++) | 28/48(58.3%) |
|  | SRP+Ro-52 | 15/48(31.3%) |
|  | SRP+PM-SCL-75 | 3/48(6.3%) |
|  | SRP+Jo-1 | 1/48(2.1%) |
|  | SRP+PL12 | 1/48(2.1%) |
| **Mean onset to medication interval(month)** | | 6（3.75，13.5） |
| **Initial treatment drugs(initial 3 months)** | Glucocorticoid N(n%) | 46（95.8%） |
|  | IVIg(combination or separately use) N(n%) | 26（54.2%） |
|  | MTX(combination) N(n%) | 21（43.8%） |
|  | CTX(combination) N(n%) | 5（10.4%） |
|  | AZA(combination) N(n%) | 3（6.3%） |
|  | tacrolimus(combination) N(n%) | 3（6.3%） |
|  | Two or more immunotherapy drugs N(n%) | 39（81.25%） |
